# Real-Time Parking Space Management System Based on a Low-Power Embedded Platform

**DOI:** 10.3390/s25227009

**Published:** 2025-11-17

**Authors:** Kapyol Kim, Jongwon Lee, Incheol Jeong, Jungil Jung, Jinsoo Cho

**Affiliations:** 1 Department of Computer Engineering, Gachon University, Seongnam 1342, Republic of Korea; kkl81@gachon.ac.kr (K.K.); 123whddnjs@gachon.ac.kr (J.L.); kaddo@gachon.ac.kr (I.J.); 2 PCT Co., Ltd., Gumi-Si 39422, Republic of Korea; ceo@powerct.kr

**Keywords:** low-power embedded, real-time video processing, object detection, YOLO, smart parking system, machine learning, video analytics, RTSP, AI

## Abstract

This study proposes an edge-centric outdoor parking management system that performs on-site inference on a low-power embedded device and outputs slot-level occupancy decisions in real time. A dataset comprising 13,691 images was constructed using two cameras capturing frames every 3–5 s under diverse weather and illumination conditions, and a YOLOv8-based detector was trained for vehicle recognition. Beyond raw detections, a temporal occupancy decision module is introduced to map detections to predefined slot regions of interest (ROIs) while applying temporal smoothing and occlusion-robust rules, thereby improving stability under rainy and nighttime conditions. When deployed on an AI-BOX edge platform, the proposed system achieves end-to-end latency p50/p95 of 195 ms and 400 ms, respectively, while sustaining 10 FPS at 3.35 W (2.99 FPS/W) during continuous 24-hour operation. Compared with conventional sensor-based architectures, the proposed design significantly reduces upfront deployment costs and recurring maintenance requirements. Furthermore, when integrated with dynamic pricing mechanisms, it enables accurate and automated fee calculation based on real-time occupancy data. Overall, the results demonstrate that the proposed approach provides a flexible, scalable, and cost-efficient foundation for next-generation smart parking infrastructure.

## 1. Introduction

Efficient parking space management has become increasingly important in recent years due to urbanization and the growing number of vehicles [1]. Existing centralized parking management systems face limitations in real-time processing and scalability, highlighting the need for a new approach [2]. In this study, we propose an outdoor parking space management system that leverages Low-Power Embedded and artificial intelligence technologies to address these challenges.

In earlier parking facilities, on-site personnel were commonly deployed to manage vehicle access and oversee daily parking operations. While this manpower-based approach required relatively low initial investment, it inevitably resulted in continuous labor costs and recurring operational expenses. Prior studies have noted that such non-automated systems often lead to substantial long-term financial burdens due to their dependence on human resources [3,4]. As parking control technologies matured, automated systems utilizing underground loop coils in addition to geomagnetic and ultrasonic sensors were introduced to detect individual space occupancy. These sensor-based approaches allowed real-time vehicle presence monitoring; however, their installation required excavation of the roadway and pavement work, leading to substantial upfront construction costs. Furthermore, recurring maintenance demands, which often involve sensor malfunctions, hardware replacement, and periodic battery service, have been cited as ongoing financial burdens in long-term operation [5,6]. More recently, intelligent parking management systems based on computer vision and artificial intelligence have emerged to address these limitations. By utilizing camera imagery, such systems can accurately determine parking occupancy, identify vehicle types and license plates, and even detect anomalous behaviors.

As a result, vision-based approaches are increasingly regarded as more flexible, scalable, and cost-efficient alternatives for modern parking management infrastructures [7,8]. In particular, by integrating visual analytics with pricing algorithms, these intelligent systems can provide more accurate and automated fee calculations while significantly reducing the deployment and maintenance costs compared to conventional sensor-based architectures. Furthermore, recent studies on dynamic pricing strategies for smart parking [9,10] have demonstrated that combining real-time occupancy data with adaptive pricing models can further enhance operational efficiency and economic sustainability. Therefore, they are considered a practical and economically advantageous foundation for developing advanced parking management solutions in this study.

Managing outdoor parking environments remains a challenging task due to diverse and dynamic conditions such as weather variations, illumination changes, and the presence of heterogeneous vehicle types [11]. These external factors often degrade the reliability of conventional monitoring approaches, making it difficult to accurately identify parking occupancy. Furthermore, centralized architectures inherently suffer from latency and scalability limitations because large volumes of video data must be continuously transmitted and processed on remote servers, resulting in performance degradation under constrained network bandwidth [12].

To overcome these challenges, this study adopts an edge-centric architecture that performs on-site inference using a low-power embedded platform. By eliminating the need to transmit raw video streams to a centralized server, the proposed system significantly enhances real-time responsiveness and strengthens data security [13]. This distributed processing approach reduces network overhead and enables instant decision-making, which is essential for intelligent parking management systems that rely on continuous video analytics [14]. In addition, by integrating YOLOv8 (an object detection model optimized for lightweight embedded environments), the system achieves high-precision vehicle detection [15] while maintaining efficient processing throughput, thereby expanding its applicability to large-scale outdoor parking scenarios [16].

The primary objectives of this study can be defined in three key aspects. First, the proposed system demonstrates that real-time performance can be achieved even on low-power embedded boards, thereby providing a cost-effective alternative to conventional centralized architectures without compromising efficiency. Second, by enabling a single edge device to manage multiple parking spaces, the system reduces maintenance overhead and lowers long-term operational costs compared to sensor-dense legacy solutions. Third, the proposed architecture ensures compatibility and scalability with existing parking management infrastructures, offering a practical and incremental migration path for modernizing traditional centralized systems. Overall, this study presents a flexible and resource-efficient system architecture and implementation methodology for next-generation outdoor parking management, providing a practical alternative to overcome the limitations of existing approaches.

The remainder of this paper is organized as follows: Section 2 reviews related research relevant to the proposed system, establishing the technical background and motivating the objectives of this study. Section 3 presents the overall system design, describing the architecture and data flow of the AI-BOX, which functions as the edge device in the proposed framework. Section 4 details the system implementation, including dataset collection procedures, the configuration of the AI-BOX execution environment, and the core design of the parking management system, along with its user interface for service delivery. Section 5 evaluates the performance of the proposed system, demonstrating that the edge-based architecture achieves real-time operation and comparable accuracy to conventional centralized systems, while significantly reducing computational resource requirements. This section also verifies the system’s continuous 24 h operability through hardware utilization measurements. Finally, Section 6 presents the conclusions and discussion.

## 2. Related Research

### 2.1. Comparison of Conventional Systems

Conventional parking management systems can be largely categorized into two types: the In-ground Sensor-based Parking System and the Gate-based Centralized Parking Management System. The former installs hardware at each parking slot to directly detect occupancy, whereas the latter concentrates devices at key entry points and performs integrated monitoring through a centralized control server. These two approaches exhibit clear differences in both initial capital expenditure (CAPEX).

#### 2.1.1. In-Ground Sensor-Based Parking System

**Figure 1:**
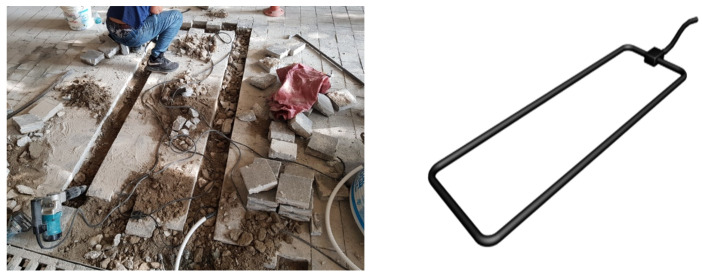
Installation process and structural characteristics of the in-ground loop coil parking surveillance system.

**Figure 2:**
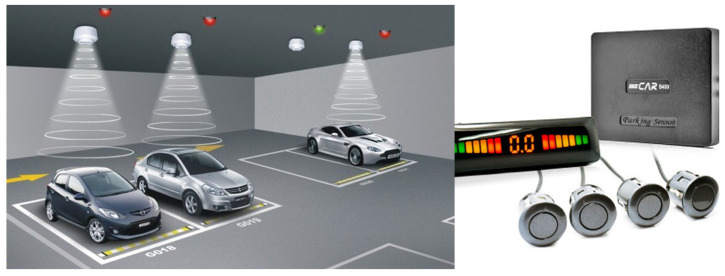
Installation example and structural details of an camera-based smart parking detection system.

The in-ground sensing approach installs hardware such as loop coils, geomagnetic sensors, and ultrasonic sensors at each parking slot or gate to directly detect the presence of vehicles [2,5]. Because the number of sensors increases in proportion to the number of parking spaces, the initial deployment cost rises significantly as the facility size grows [3]. In addition, continuous maintenance is required due to hardware aging, battery depletion, and environmental factors such as moisture and temperature fluctuations, which ultimately lead to high operational expenses (OPEX) [7]. Despite these drawbacks, this approach is still widely employed because it provides highly reliable real-time occupancy information under diverse illumination and weather conditions [6]. Specifically, as shown in Figure 1, loop-coil and geomagnetic in-ground sensors are embedded beneath the pavement to detect changes in metallic mass, whereas ultrasonic-based systems, illustrated in Figure 2, are installed above the parking slot or on a front pillar to measure reflected signals for occupancy determination. These structural differences directly affect maintenance procedures and installation complexity, resulting in varying cost-efficiency characteristics depending on the deployment environment [5].

#### 2.1.2. Camera-Based Parking Management System

**Figure 3:**
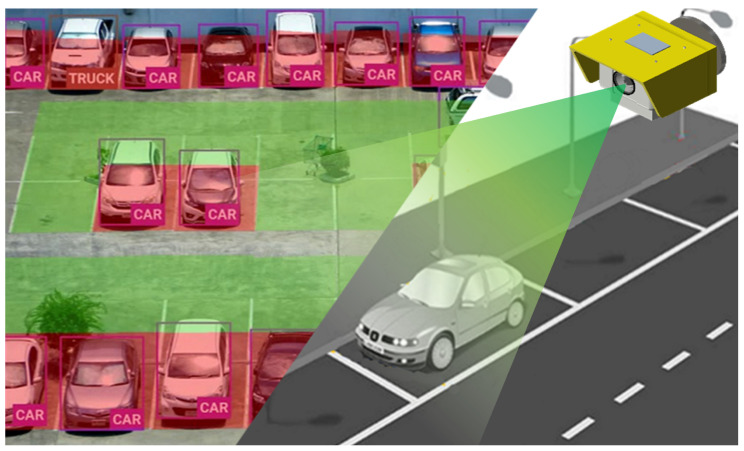
Installation example and structural details of an ultrasonic sensor-based parking detection system.

An example of a camera-based centralized system is shown in Figure 3. The camera-based centralized system deploys CCTV or IP cameras at strategic viewpoints to monitor multiple parking spaces and transmits video streams to a central control server, where occupancy inference is performed [1,6]. Because only a limited number of cameras are required to cover a parking zone, the initial CAPEX is lower than slot-level sensor deployments, and maintenance is largely confined to camera cleaning, calibration, and network upkeep [7]. However, the accuracy of this approach can be affected by illumination, weather, and occlusion [11], and real-time performance may be degraded under network congestion or server overload [2]. Moreover, as inference is performed at a centralized server, scalability is constrained by bandwidth and compute capacity, making it less efficient for large installations that require continuous per slot occupancy monitoring [8].

#### 2.1.3. Comparison of Deployment and Maintenance Costs in Conventional Systems

Table 1 provides a comparative overview of representative parking occupancy monitoring approaches with respect to their deployment costs and operational characteristics. In-ground sensor-based systems require the installation of a dedicated sensing module at each parking slot, leading to capital expenditures that scale proportionally with facility size. While these systems offer robust and weather-resilient detection performance, their hardware-intensive configuration also results in the highest construction effort and installation complexity among the examined methods. On-surface IoT sensors can reduce construction overhead; however, their dependence on battery-powered devices inevitably increases maintenance frequency and long-term service requirements.

Conversely, camera-based centralized systems offer lower initial deployment costs, as a single camera can monitor multiple parking spaces within its field of view. Nevertheless, their reliance on a central processing server introduces inherent scalability limitations, where both inference latency and detection reliability may degrade under constrained network bandwidth or heavy computational workloads.

Overall, the comparison reveals a clear trade-off in conventional approaches: although they are capable of delivering reliable occupancy monitoring, their cost structure, maintenance burden, and limited scalability remain significant obstacles for large-scale or high-density parking environments. These limitations underscore the necessity of a distributed, edge-centric architecture that preserves the benefits of vision-based monitoring while mitigating network dependency and reducing long-term operational overhead.

### 2.2. Object Recognition Algorithm

Object recognition algorithms have advanced rapidly since AlexNet won the ImageNet Challenge in 2012 [21]. Since then, many researchers have proposed various models based on Convolutional Neural Networks (CNNs), which, by 2015, had surpassed human recognition capabilities.

The growing need for real-time object recognition has led to the development of the YOLO algorithm [22]. YOLO detects objects by processing the entire image in a single network pass, demonstrating excellent performance in real-time object recognition. Compared to other models, YOLO strikes a better balance between speed and accuracy, operating efficiently in resource-constrained environments, such as edge devices.

In addition, lightweight models such as MobileNet, SqueezeNet, and ShuffleNet have emerged to operate efficiently in resource-constrained environments [23,24,25]. MobileNet employs depthwise separable convolution to achieve a lightweight design, while SqueezeNet focuses on reducing the number of parameters. ShuffleNet optimizes performance through channel shuffling techniques. However, despite the advancements of these lightweight models, YOLO remains recognized as the more powerful option for real-time object detection.

YOLOv8 employs an anchor-free approach, predicting bounding box coordinates directly without the need for anchor boxes. This advancement greatly enhances computational efficiency and removes the traditional computational burden associated with anchor box methods. When trained on the Common Objects in Context (COCO) dataset, which includes 80 object classes, using the YOLOv8n model on an NVIDIA GeForce RTX 3090 GPU, YOLOv8 achieved a mean average precision (mAP) of 50.1 and completed detections in just 2 milliseconds (ms), demonstrating higher accuracy and faster processing speeds than existing object recognition algorithms [26,27].

**Figure 4:**
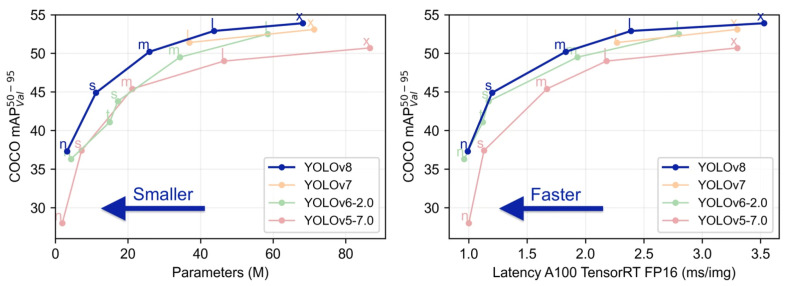
YOLOv 8 Performance Comparison Graph (Adapted from [25]).

The benchmark test illustrated in Figure 4 was conducted on an NVIDIA GeForce RTX 3090 GPU, using images primarily set to a resolution of 640 × 640. The results indicate that YOLOv8 models exhibited significant improvements in both accuracy and speed compared to previous YOLO models.

In YOLOv8, bounding boxes are an essential tool for predicting the locations of objects. Each bounding box specifies the coordinates of the object’s center, along with its width and height, represented as x, y, w, and h.

**Figure 5:**
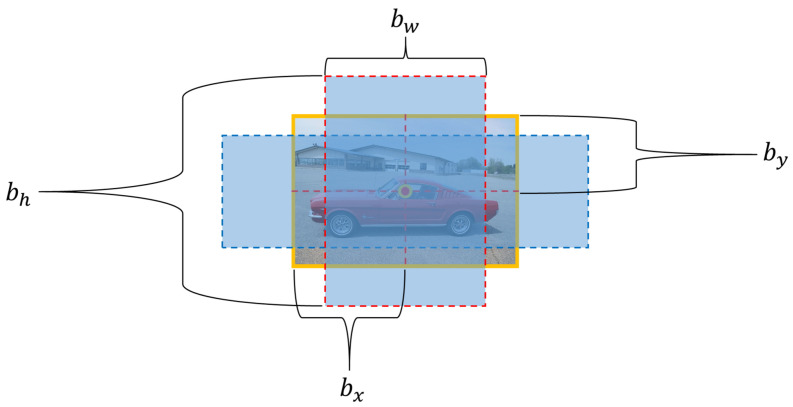
YOLO Bounding Box Structure.

In Figure 5 above, bx and by represent the center coordinates of the bounding box, while bw and bh denote the width and height of the object, respectively. This method is consistent in YOLOv8, allowing for an accurate calculation of an object’s position and size within an image. In YOLOv8, the bounding box coordinates are estimated using (1) [26].

(1) $$\begin{array}{cc} x & =\sigma \left(t_{x}\right)+c_{x},\\ y & =\sigma \left(t_{y}\right)+c_{y},\\ w & =e^{t_{w}},\\ h & =e^{t_{h}} \end{array}$$

In Equation (1), *x* and *y* represent the center coordinates of the bounding box within a grid cell, computed using the sigmoid function σ as shown in Equation (2) [28]. These coordinates are obtained from the grid cell offsets $c_{x},c_{y}$ plus the predicted terms $\sigma (t_{x})$ and $\sigma (t_{y})$.

(2) $$\mathrm{sigmoid}\left(x\right)=\sigma \left(x\right)=\frac{1}{1+e^{-x}}$$

*w* and *h* represent the width and height of the bounding box, respectively, with the exponential function applied to transform the values of $t_{w}$ and $t_{h}$. The variables $t_{x},t_{y},t_{w},t_{h}$ are the values predicted by the model during training, while $c_{x}$ and $c_{y}$ denote the coordinates of the corresponding cell. This approach enables YOLOv8 to predict object positions more accurately without relying on anchor boxes. By adopting an anchor-free methodology, YOLOv8 addresses the challenges associated with sizing anchor boxes in YOLOv3 and YOLOv4, facilitating more efficient operations.

In YOLOv8, the probability of object detection is calculated using Equation (3) below.

(3) $$\mathrm{Pr}\left({\mathrm{Class}}_{i}|\mathrm{Object}\right)\times \mathrm{Pr}\left(\mathrm{Object}\right)\times \mathrm{IoU}\frac{\mathrm{truth}}{\mathrm{pred}}=\mathrm{Pr}\left({\mathrm{Class}}_{i}\right)\times \mathrm{IoU}\frac{\mathrm{truth}}{\mathrm{pred}}$$

In Equation (3), $\mathrm{Pr}({\mathrm{Class}}_{i}|\mathrm{Object})$ denotes the probability that a specific detected object belongs to class *i*. This represents the likelihood that an object (e.g., a human, car, etc.) is classified into a particular category when detected.

For example, if we assume that within one bounding box of a grid cell, $\mathrm{Pr}({\mathrm{Class}}_{i}|\mathrm{Object})=0.7$, Pr(Object)=0.8, and $\mathrm{IoU}\frac{\mathrm{truth}}{\mathrm{pred}}=0.9$, then the final probability that the given object belongs to that class in that bounding box is P=0.7×0.8×0.9=0.504, the probability that the object belongs to the class is 50.4%.

The reasons for selecting the YOLOv8 model in this paper are as follows:

- Lightweight Nano-Model: The YOLOv8 nano-model features a lightweight architecture that can be efficiently executed on edge devices, making it well-suited for environments with limited memory and computational resources.
- Real-time processing: Fast inference speeds enable real-time vehicle detection, allowing for quick monitoring of parking lot conditions.
- Edge Device Compatibility: It is easily adaptable to various programming languages, including Python, and meets the performance requirements of edge devices.

In this paper, we utilized the YOLOv8 algorithm to design a system for real-time vehicle detection in outdoor parking spaces. Additionally, we trained the model to analyze aerial view images taken from a height of over 15 m, allowing for the simultaneous monitoring of at least 20 parking spaces.

YOLOv8 was selected for this study because it provides an optimal balance between detection accuracy, inference speed, and hardware compatibility on embedded platforms. While more recent studies have explored advanced lightweight architectures for edge computing, such as MobileDet, Edge TPU-based frameworks, YOLOv10, and Edge Former, these models primarily target specialized hardware or remain in early experimental stages [29,30,31,32]. Therefore, YOLOv8 was considered the most suitable and stable model for real-world deployment on low-power edge devices in this work.

### 2.3. Web-Based Control System Technology - Asynchronous Web Framework

Asynchronous web frameworks can efficiently handle multiple requests simultaneously, significantly improving the performance of input/output (I/O) bound operations [33]. AIOHTTP is a representative asynchronous HyperText Transfer Protocol (HTTP) client/server framework based on Python’s asyncio library [34]. This framework utilizes asynchronous request processing with coroutines to make efficient use of system resources [35]. Table 2 presents a performance comparison between AIOHTTP and other Python web frameworks.

As shown in Table 2, AIOHTTP demonstrates high request throughput and low response times compared to other Python web frameworks. This indicates that the asynchronous processing of AIOHTTP is well-suited for implementing high-performance web applications.

In this study, we utilized AIOHTTP to asynchronously process HTTP requests, including monitoring parking lot status, changing settings, and sending real-time notifications. This approach allowed us to handle multiple client requests simultaneously while maintaining the system’s responsiveness.

## 3. Proposed System Design

### 3.1. Proposed System Overview

**Figure 6:**
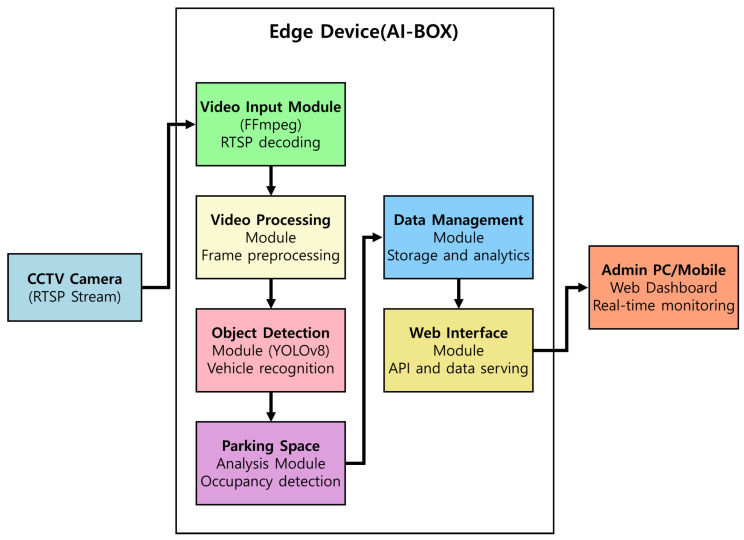
Proposed System Architecture.

The edge device-based outdoor parking space management system proposed in this paper is an integrated solution that leverages real-time image processing and artificial intelligence technology to support the efficient operation and management of parking lots. Figure 6 illustrates the overall architecture of the proposed system, which comprises CCTV(Closed-Circuit Television) cameras, edge devices, and an administrator’s personal computer (PC) or mobile dashboard. Installed onsite at the parking lot to perform real-time video processing and analysis. In this system, the edge device, referred to as AI-BOX, has several main modules, the functions of which are presented in Table 3.

The proposed system combines edge computing technology with the latest object recognition algorithms to overcome the limitations of traditional centralized systems, providing a more efficient and scalable parking management solution. The edge device (AI-BOX) processes video data in real-time onsite, ensuring timeliness and reducing network load.

During the development process, several implementation strategies were tested to achieve real-time performance under low-power embedded constraints. Initial prototypes based on Open Source Computer Vision Library (OpenCV) pipelines suffered from high Central Processing Unit (CPU) load and frequent frame drops, which made continuous streaming and inference unstable. Replacing this structure with an independent Fast Forward Motion Picture Experts Group (FFmpeg) decoding process reduced computational overhead and stabilized frame processing throughput.

For inter-process communication, multiple Message Queue (MQ) systems such as RabbitMQ, Redis Queue, and Apache Kafka were evaluated. While these platforms are efficient in high-performance cloud environments, they generated excessive CPU and memory overhead under continuous RTSP streaming and caused latency spikes on the embedded board. Database-based communication produced similar Input/Output (I/O) bottlenecks. To address these issues, a lightweight folder-based queuing mechanism was developed, enabling direct data exchange between processes through the local file system without database access or MQ middleware. This design eliminated unnecessary context switching and resource contention, improving both reliability and throughput.

Another critical bottleneck occurred in the web communication layer. Conventional Hypertext Transfer Protocol (HTTP)-based data transfer caused a rapid increase in hand-shake and polling requests when multiple monitoring clients accessed the system, occasionally freezing the process. To overcome this limitation, an asynchronous event-driven framework combining Asynchronous Input/Output for HTTP (aiohttp) and Socket Input/Output (Socket.IO) was implemented, enabling bidirectional communication between the AI-BOX and the web dashboard without continuous HTTP polling. This approach removed handshake overload, reduced I/O blocking, and ensured stable real-time synchronization. In terms of model selection, several object detection frameworks were evaluated, including Faster Region-Convolutional Neural Network (Faster R-CNN), Single-Shot Multi-Box Detector (SSD), and MobileNet-based models. However, these architectures were either computationally heavy or exhibited lower detection accuracy in small object and occlusion scenarios. YOLOv8 was adopted because it provides a balanced trade-off between inference speed, accuracy, and model size, particularly in its Nano configuration, which is optimized for edge deployment. The model’s decoupled detection head and anchor-free architecture further reduced post-processing latency, allowing real-time performance on the 7–8 W embedded hardware. Instead of modifying the YOLOv8 network itself, this study focused on designing an optimized data pipeline and asynchronous system architecture to fully exploit the model’s lightweight characteristics within the AI-BOX platform. Through these iterative trials and architectural refinements, the proposed system achieved reliable low-latency operation within a 7–8 W embedded power budget, demonstrating clear system-level innovation for edge-based intelligent parking management.

### 3.2. System Configuration and Key Module Design for Parking Recognition

**Figure 7:**
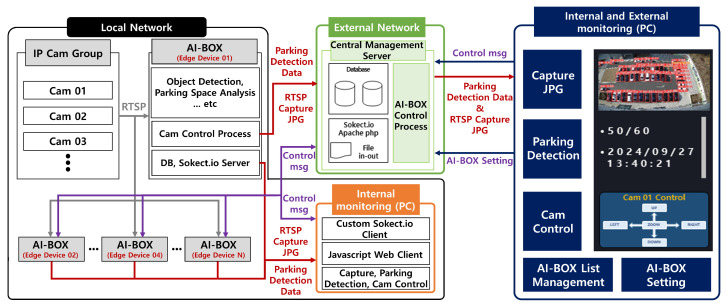
Edge Device-Based Outdoor Parking Space Control System Architecture.The number of AI-BOX units can be scaled according to the number of parking zones, and the CAM modules correspond to the number of cameras installed in the parking area. The red arrows indicate the transmission of detection coordinates and captured images via RTSP, the purple arrows represent the messaging paths used for controlling each module, and the blue arrows denote the routes through which upper-level control messages and AI-BOX configuration commands are delivered.

Following the implementation and optimization strategies described in Section 3.1, this section details the configuration and key modules of the edge device (AI-BOX)–based outdoor parking space management system. Figure 7 illustrates the block diagram of the entire system.

The proposed system is broadly divided into a local network and an external network, as illustrated in Figure 7. The local network consists of on-site components installed in the parking lot, including a group of IP cameras and an AI-BOX. The AI-BOX, based on an embedded platform, serves as the core device of the system, performing real-time image processing and parking lot analysis. It captures and stores video streams from IP cameras using the RTSP and determines the occupancy status of parking spaces through its internal Object Detection Module and Parking Space Analysis Module. Additionally, it controls camera parameters through the Camera Control Process and manages communication using the built-in database (DB) and Socket.IO server.

For parking zone definition, the administrator dashboard utilizes polygon.js, which provides an intuitive graphical interface to configure and manage parking zones. These features collectively enhance the flexibility and user-friendliness of the system operation.

### 3.3. Parking Space Analysis Module Detailed Design

Object Detection processes incoming video streams in real time using a YOLOv8-based vehicle detector, extracting bounding boxes, class labels, and confidence scores for each frame. Through preprocessing, confidence filtering, and Non-Maximum Suppression, only the most reliable detections are retained and rescaled to the original image dimensions. The finalized detection outputs are then forwarded to the Parking Space Analysis Module as the primary input for determining slot-level occupancy, forming the basis for downstream parking recognition. The Parking Space Analysis Module evaluates the occupancy status of parking spaces using the vehicle object information recognized by the Object Detection Module. Its main functions and algorithms are as follows.

Definition of key variables in the Parking Space Analysis Module:

- **parkingSpaces**: an array of parking space information, where each element contains the ID, coordinates, status, etc.;
- **detectedVehicles**: an array containing information about the detected vehicle objects;
- **occupancyMap**: a map representing the occupancy status of parking spaces;
- **IOU_THRESHOLD**: intersection over Union (IoU) threshold used to determine if a vehicle has occupied a parking spot;
- **TEMPORAL_WINDOW**: the number of frames used for temporal filtering;
- **violationThreshold**: a time threshold (in seconds) used to determine parking violations.

**Figure 8:**
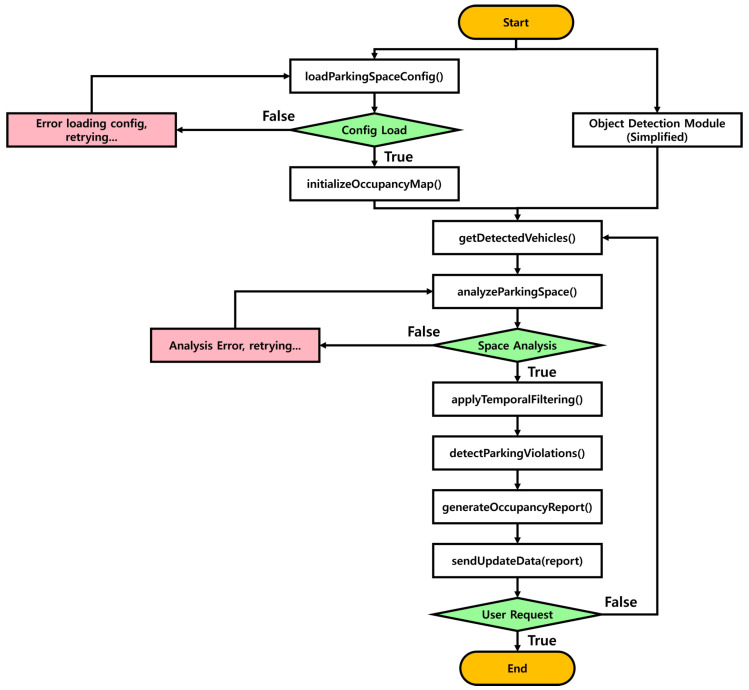
Flowchart of the Parking Space Analysis Module.

Figure 8 shows a detailed flowchart of the Parking Space Analysis Module.

The Parking Space Analysis Module begins by loading the parking space configuration data, which is saved by the web administrator in the database, through an SQL (Structured Query Language) query that retrieves information from the parking spaces table. Using this data, the module initializes the occupancy map and sets the initial status of each parking space to empty. The module then receives vehicle detection results from the Object Detection Module, including bounding box coordinates and confidence scores. For each parking space, the system calculates the center point from its coordinates and checks whether this point overlaps with any detected vehicle bounding box. The overlap is determined using the calculate IoU function, which computes the Intersection over Union between the parking space center and the vehicle bounding box. If the IoU exceeds the predefined threshold, the corresponding parking space is marked as occupied in the occupancy map, and the updated status is stored accordingly.

To ensure robust results, temporal filtering is applied by tracking the history of each parking space across the defined temporal window of frames, with the final state determined by majority voting. The system also monitors the occupancy duration of each parking space to detect violations. When the occupancy time exceeds the violation threshold, the corresponding space is added to the violation list. Finally, the module generates a comprehensive occupancy report summarizing the current overall parking lot status and any violations, and this report is sent to the main server to update the database.

Through the integration of the Parking Space Analysis Module and Object Detection Module, along with their parallel processing capabilities, the proposed system offers an efficient and scalable parking management solution. By leveraging the advantages of real-time processing within the local network and centralized management via an external network, the system achieves flexibility suitable for parking facilities of various sizes. Additionally, the web-based management interface enhances camera control and parking lot zoning, significantly improving the system’s operational and maintenance efficiency.

## 4. Proposed System Implementation

### 4.1. Proposed System Implementation Environment and Device Fabrication

For the system configuration proposed in this paper, a micro PC was used as the hardware for the AI-BOX, and its detailed specifications are shown in Table 4.

For the operating system of the AI-BOX, we utilized Linux kernel 5.10 (Long-Term Support, LTS), which is widely recognized for its stability and proven compatibility with Python-based AI frameworks. During system integration, newer kernel versions such as 6.x caused dependency and driver conflicts with the Ultralytics YOLOv8, AIOHTTP, and python-socketio libraries, resulting in unstable RTSP streaming and event-loop behavior. Therefore, kernel 5.10 was selected as a stable and validated environment for real-time edge inference. Future studies will benchmark kernel 6.x once driver and library dependencies become fully supported. The development environment was established using Python 3.7 virtual environments to ensure package isolation, with core libraries including OpenCV, YOLOv8, NumPy, python-socketio, and AIOHTTP, while FFmpeg was executed as a separate process for RTSP stream handling.

### 4.2. Outdoor Parking Lot Dataset Construction

A dataset was constructed to record the parking and exit status of vehicles in the outdoor parking lot of Gachon University, facilitating the collection of parking and exit data. This dataset serves as training data for recognizing vehicles in outdoor parking environments and for developing parking and exit detection technology, ultimately aimed at enhancing the performance of the parking area monitoring system.

The data collection process is as follows:

- Photographing the outdoor parking lot at Gachon University Global Campus;
- Conducting photography from June 2023 to December 2023 (over 7 months);
- Positioning cameras to capture the entire parking lot;
- Periodically photographing in various environmental conditions, including different weather and times of day;
- Collecting a total of 13,691 images featuring specific environmental characteristics.

**Figure 9:**
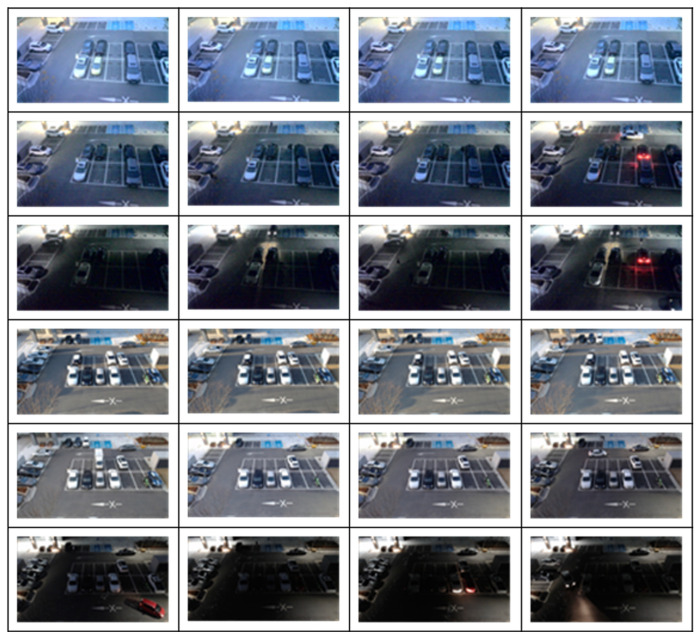
Example of Outdoor Parking Lot Data Collection.

Figure 9 below presents a subset of the obtained dataset.

To enhance the quality of the collected data and filter only the data suitable for the YOLOv8 training dataset, we conducted a cleansing operation.

The cleansing operation was carried out in several systematic stages to ensure that only high-quality and diverse images were included in the YOLOv8 training dataset:

- Duplicate and corrupted data removal: RTSP stream collection occasionally produced damaged or duplicated frames due to network latency or packet loss. These corrupted or identical images were automatically detected and removed using a custom Python script.
- Frame selection criteria: To avoid redundant scenes, frames were extracted at regular time intervals (approximately 3–5 s), ensuring a balanced distribution of illumination, viewing angle, and vehicle occupancy across samples.
- Preliminary detection-based filtering: A pretrained YOLOv8 model was used to per-form preliminary detection. Frames with detection confidence below 0.5 were discarded to exclude low-quality or ambiguous samples.
- Image quality filtering: Blurred or poorly illuminated images caused by motion, rain, or low brightness were filtered out using the variance of Laplacian and histogram-based brightness thresholds.
- Region validation: Each remaining frame was checked to confirm that vehicle objects were located within the defined polygonal parking zones annotated in the dataset.
- Manual verification and correction: After automated filtering, all remaining images were manually reviewed to remove frames where vehicles were partially occluded, misaligned with parking lines, or affected by reflections or camera artifacts.

After this cleansing process, 9922 high-quality images remained from the original 13,691 collected images, which were later augmented to 13,247 images for training.

This screened data encompassed a variety of parking situations, vehicle types, and lighting conditions, ensuring robust model performance in real-world environments. The dataset information for classifying the final vehicle objects is presented in Table 5.

As shown in Table 5, data augmentation resulted in a total of 13,247 images from the initial 9922 datasets. This dataset contains approximately 55,000 labels, strategically chosen to ensure the model’s performance across various environments. The resulting dataset was used to train the YOLOv8n model, ensuring that the model can efficiently recognize objects in edge computing environments.

### 4.3. Outdoor Parking Lot AI Model Training

The YOLOv8n model was trained using the Ultralytics framework with a configuration optimized for edge deployment. Training was performed on a system equipped with an NVIDIA GeForce RTX 2080 GPU. The training process followed a supervised learning pipeline using stochastic gradient descent (SGD) with early stopping to prevent overfitting. Table 6 summarizes the main hyperparameters used for training.

These hyperparameters were tuned to balance inference accuracy and computational efficiency, ensuring real-time performance of the YOLOv8n model under the 7–8 W embedded constraint.

### 4.4. AI-BOX Implementation

AI-BOX is a core system based on edge computing for outdoor parking lot management, enabling efficient parking space management and real-time monitoring. This section outlines the software structure of AI-BOX, its main modules, and the implementation of its core logic.

#### 4.4.1. AI-BOX (Edge Device) Software System Architecture

**Figure 10:**
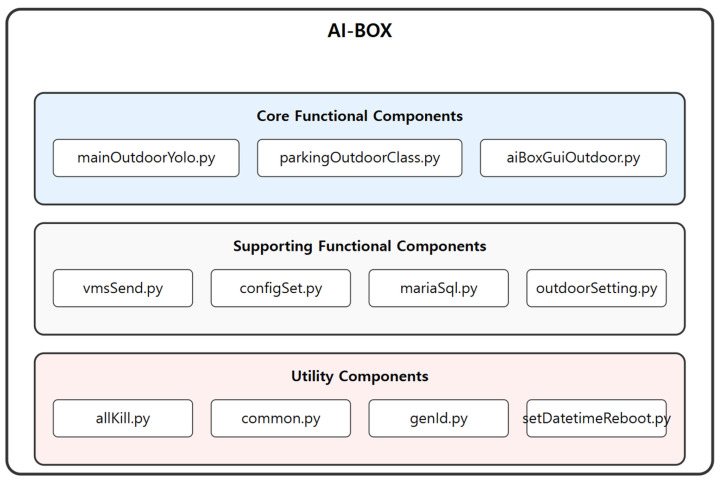
AI-BOX Software Architecture.

The AI-BOX software is designed with a three-tier hierarchical structure, where Python modules handle the system’s various functions at each layer. Specifically, the mainOutdoorYolo.py and parkingOutdoorClass.py modules perform core tasks for parking control, such as object recognition, parking and exit detection, internal and external communication, and process management. Figure 10 below illustrates the AI-BOX software hierarchy.

The core functional modules of AI-BOX consist of mainOutdoorYolo.py, parkingOutdoorClass.py, and aiBoxGuiOutdoor.py. mainOutdoorYolo.py controls the overall system, manages communication with external systems, and provides the web-based control interface. It also monitors processes and automatically restarts them in the event of abnormal termination. parkingOutdoorClass.py performs essential tasks such as video input, preprocessing, object detection, and parking space analysis, incorporating the necessary submodules. aiBoxGuiOutdoor.py provides the graphical user interface (GUI).

The supporting functional modules include vmsSend.py, configSet.py, mariaSql.py, and outdoorSetting.py. These modules handle event transmission, system configuration management, database integration and query processing, and system parameter management, respectively.

The utility modules comprise allKill.py, common.py, genId.py, and setDatetimeReboot.py. They provide functionalities for process termination, definition of common functions and variables, client ID generation, and time configuration management.

The AI-BOX software architecture is designed to efficiently meet the diverse requirements of a parking management system. Each module is optimized to ensure real-time management, ease of maintenance, and stable operation within parking environments, providing a foundation for sustained reliability and performance. With these structural strengths, AI-BOX delivers reliable and efficient parking control solutions across a wide range of environments.

#### 4.4.2. Core Module Implementation: parkingOutdoorClass.py

The parkingOutdoorClass.py module implements the core functionality of AI-BOX and consists of four main components:

- Video Input Module: Captures images from the RTSP stream using FFmpeg and stores them in the frame folder.
- Video Processing Module: Preprocesses the stored images with OpenCV and improves quality through techniques such as sharpening and histogram equalization.
- Object Detection Module: Utilizes the YOLOv8n model, trained with the proposed system, to detect vehicle objects in preprocessed images. It extracts bounding box coordinates and confidence scores of the detected objects.
- Parking Space Analysis Module: Loads parking lot coordinate data from JSON(JavaScript Object Notation) files, calculates the center coordinates of each parking space, and determines parking status by comparing these centers with the bounding boxes of detected vehicles. It also computes both the overall occupancy rate and the status of individual parking spaces.

**Figure 11:**
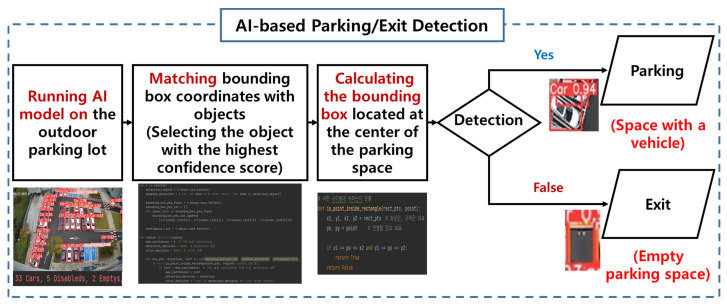
Parking/Exit Detection Logic.

Figure 11 clearly illustrates the park/exit decision logic of the proposed system. The figure shows the sequential process: running the AI model on the outdoor parking lot, matching bounding box coordinates with detected objects, and calculating whether a bounding box overlaps the center of a parking space. Based on this logic, the system determines whether the space is occupied (Parking) or empty (Exit), enabling real-time monitoring of parking and exit conditions.

### 4.5. Monitoring Web Service Implementation

The AI-BOX control web service is designed to efficiently manage and monitor parking lots through a user-friendly interface. This section outlines the main screens and functionalities of the control web service.

To ensure security and data privacy, the AIOHTTP-based monitoring web interface applies Hypertext Transfer Protocol Secure/Transport Layer Security (HTTPS/TLS) encryption for all communications and implements session-based authentication to prevent unauthorized access. User sessions automatically expire after a defined period of inactivity, and role-based access control restricts administrative operations to authorized users only. Communication between each AI-BOX device and the administrator dashboard occurs through encrypted local or Virtual Private Network (VPN) channels, while data ex-change with the central server follows a secured Representational State Transfer (RESTful) Application Programming Interface (API) protocol. All API requests include authentication tokens in the Hypertext Transfer Protocol (HTTP) header to verify identity and enforce access control. In accordance with general data protection principles (e.g., General Data Protection Regulation, GDPR), the system does not process any personally identifiable in-formation. Only non-personal operational data, such as vehicle numbers, detection results, and captured images, are transmitted to the central server for parking management purposes. These data are handled under strict access authorization and are not shared with third parties.

#### 4.5.1. Screens and Features of the Monitoring Web Service

The AI-BOX control web service is implemented using the AIOHTTP web framework, Socket.IO, and Jinja2 templates. It provides functionalities such as parking and exit monitoring, camera settings, and parking zone configurations across nine screens. Table 7 summarizes the main functions of each screen within the control web service.

As outlined in Table 7, the AI-BOX control web service offers various functions to enhance the efficiency of parking lot management. The features available on each screen facilitate real-time monitoring, system settings, event management, and more. With these capabilities, AI-BOX significantly improves the reliability and effectiveness of parking control.

#### 4.5.2. Parking Space Settings Implementation

**Figure 12:**
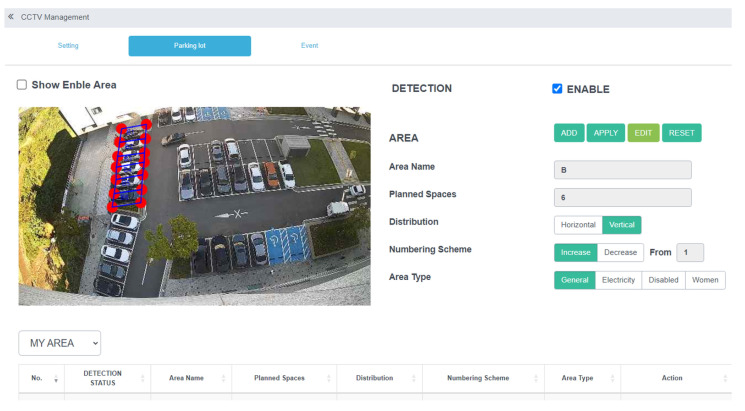
Parking Space Settings Screen Implementation. The red circles represent the four corners of the parking space, and the blue squares represent its four edges.

The functionality of the parking lot settings screen was developed using the polygon.js library. Users can add, modify, and delete parking spaces in real time and easily set information such as name, number, and direction for each space. These features empower users and administrators to efficiently manage parking spaces and configure settings for various parking environments. Figure 12 below shows a captured image of the implemented parking space settings screen.

The parking space configuration feature of the proposed system simplifies the definition of parking spaces with complex shapes and supports a wide variety of configurations, providing users with flexibility. Multiple parking spaces can be drawn and integrated on a single screen, while the automatic alignment function ensures efficient placement without interference between spaces. Additionally, the system detects conflicts among configured parking spaces, assisting users in correct placement. Users also have the option to reuse previously drawn areas, greatly enhancing convenience.

## 5. Proposed System Evaluation

### 5.1. Performance Evaluation of the Outdoor Parking Recognition Model

In this study, a recognition model specialized for outdoor parking lot environments was developed based on YOLOv8n. To objectively evaluate the model’s performance, a separate test dataset was constructed that was not used for training or validation. This test dataset consists of 2225 images of occupied parking spaces and 2189 images of empty parking spaces, totaling 4414 images. It includes images captured under various environmental conditions (snow, rain, sunny, cloudy, etc.).

**Figure 13:**
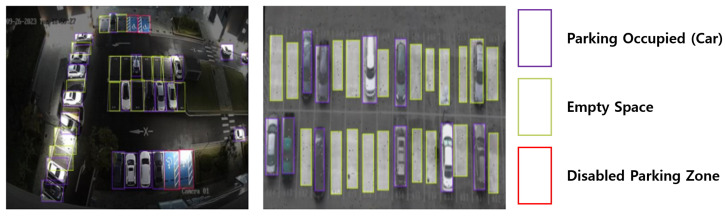
Examples of Occupied, Vacant, and Accessible Parking Spaces.

Figure 13 displays a captured image from the test data. Based on the test dataset, various performance indicators of the model, such as accuracy, precision, and recall, were analyzed.

To evaluate the proposed system, we report accuracy and latency measurements obtained directly from the training logs and single-image benchmarks of the YOLOv8 model. For comparative assessment, YOLOv12, the most recent version available at the time of writing, was included as a reference under the same experimental protocol. To ensure operation in the proposed low-power embedded environment, model parameters were adjusted to apply lightweight versions of each model.

**Figure 14:**
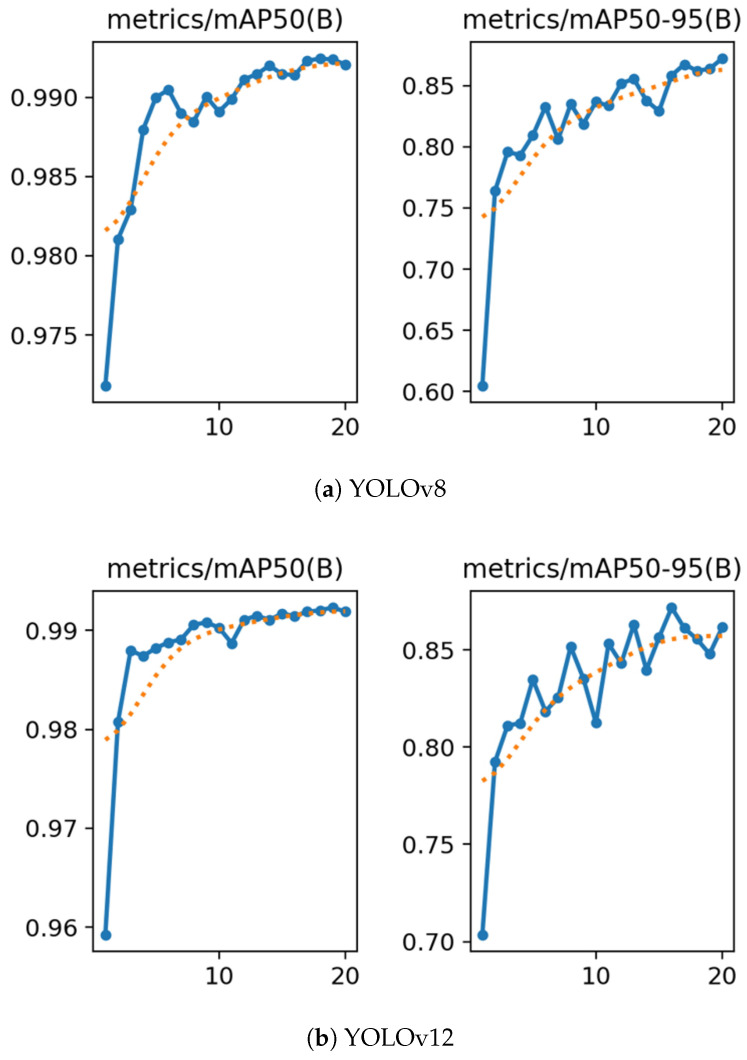
Comparison of training performance between YOLOv8 (**a**) and YOLOv12 (**b**) over 20 epochs. The blue solid curve represents the mAP50 values measured at each epoch, and the orange dotted curve represents the trend obtained through moving average smoothing.

The learning curves, as shown in Figure 14, demonstrated stable convergence for both YOLOv8 and YOLOv12. At the final epoch, the test metrics, as presented in Table 8, show that both models achieved mAP@0.5 values above 0.99 and mAP@0.5:0.95 values above 0.87. Overall, high precision was maintained, and recall remained close to the upper envelope, indicating consistent discrimination between occupied and empty spaces.

**Figure 15:**
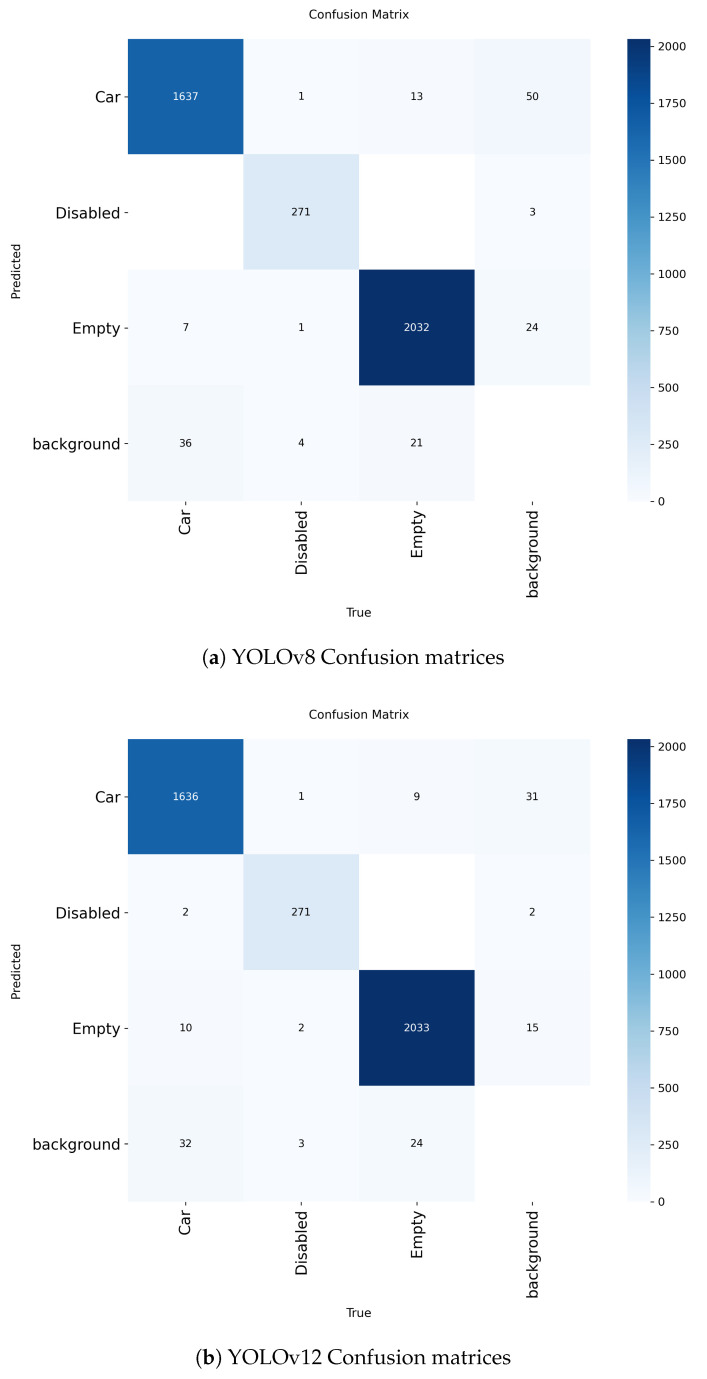
Confusion matrices of the test results for YOLOv8 (**a**) and YOLOv12 (**b**).

In addition to the quantitative metrics reported in Table 8, Figure 15 presents the confusion matrices for YOLOv8 and YOLOv12. Both models demonstrate clear discrimination among classes, with the majority of instances correctly classified. Only a small number of misclassifications occurred, primarily between visually similar categories, further confirming the reliability of the proposed system in real-world parking scenarios.

Table 9 presents the benchmark results of YOLOv8 and YOLOv12 after excluding the first 50 runs to remove initialization bias. The results are analyzed by decomposing them into preprocessing, inference, postprocessing, total latency, and the number of detections. YOLOv8 recorded an average inference time and total processing time of 226.81 ms, showing a 30 ms (≈11.6%) faster performance compared to YOLOv12 at 256.67 ms. In contrast, YOLOv12 exhibited slightly shorter postprocessing latency and demonstrated a more stable upper bound in maximum latency, reaching 485.39 ms compared to YOLOv8 is 504.05 ms. Both models maintained an identical average of 300 detections per image.

**Figure 16:**
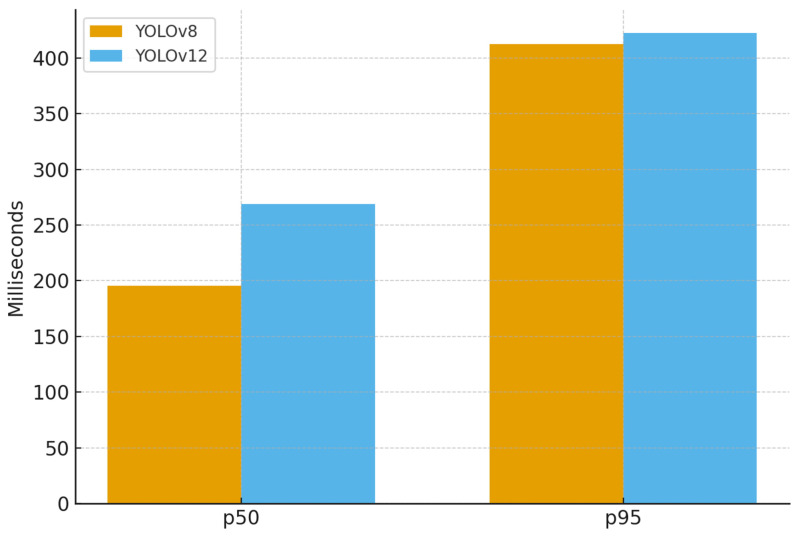
Latency distribution of YOLOv8 and YOLOv12 at p50 and p95.

Figure 16 visualizes the latency distribution based on p50 (median) and p95 (95th percentile). At the median level, YOLOv8 recorded approximately 195 ms, which is about 73 ms faster than YOLOv12 (268 ms), corresponding to a 27% improvement. Additionally, at the p95 level, both models converged above 400 ms, with YOLOv12 at approximately 415 ms and YOLOv8 at approximately 405 ms, resulting in only a 10 ms (≈2.5%) difference. These results indicate that YOLOv8 provides faster inference under typical conditions, while YOLOv12 maintains more consistent performance in higher latency ranges.

**Figure 17:**
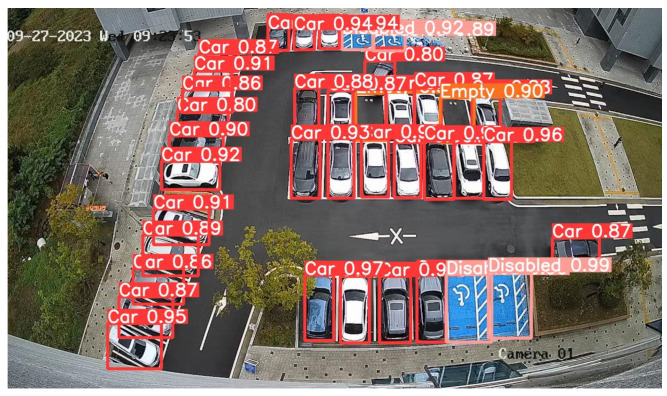
Real-time Recognition Status of Outdoor Parking Spaces at Gachon University.

In addition to the benchmark and quantitative evaluation results, the proposed system was also validated in a real-world environment. Specifically, during field tests conducted in the outdoor parking lot at Gachon University, the learning model accurately recognized vehicles, empty parking spaces, and designated disabled parking spaces. Figure 17 presents a real-time capture of the recognition status in the outdoor parking area, further illustrating the practical applicability of the system.

As a result of the performance evaluation of the outdoor parking lot recognition model, it was confirmed that the developed model demonstrated stable and high accuracy across various parking situations and vehicle types, proving to be highly effective when applied to an actual parking management system. This confirms that the proposed system is a reliable solution for vehicle recognition and parking surface management in outdoor environments.

### 5.2. Evaluation of System Resource Usage

The evaluation of system resource usage involved measuring the resource consumption of AI-BOX during real-time parking space monitoring, with the proposed system operating under conditions similar to the actual environment. The evaluation focused on three main indicators: CPU usage, memory usage, and CPU temperature, with the results presented in Table 10 below.

**Figure 18:**
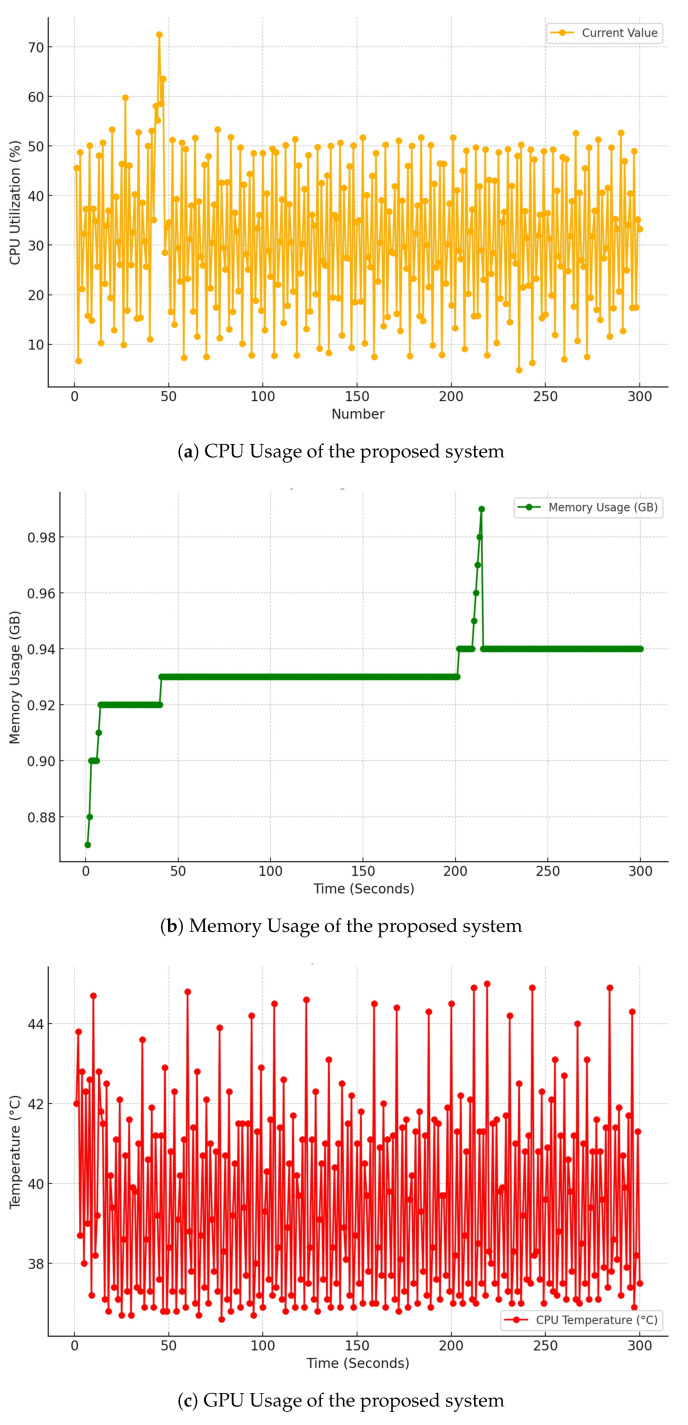
Resource Usage Evaluation of the Proposed System.

As shown in Figure 18a above, the CPU usage of AI-BOX in the proposed system averaged 31.5% over 300 seconds, representing an increase of 28.3%p from the default state of 3.2%. A temporary spike in CPU usage was observed, reaching up to 72.5%, which is estimated to occur during intensive operations of the artificial intelligence model. Notably, this fluctuation in CPU usage coincided with the activation of the image analysis algorithm. However, for the majority of the evaluation period, CPU usage remained below 30%, indicating that the system is effectively managing resources in a stable manner.

Although direct power measurement was not performed, the power consumption of the AI-BOX can be reasonably inferred from the measured CPU utilization and the hardware platform specifications. According to the official documentation of the Raspberry Pi 4 Model B, the device typically consumes about 7–8 W under full CPU load. Given that the average CPU utilization of the proposed system was approximately 31.5 %, the corresponding power consumption is estimated to be around 4–5 W during normal real-time operation. This estimation aligns with independent benchmark data [39] and confirms that the system operated stably within a low-power budget. In contrast, conventional GPU-based or cloud-dependent vision systems typically consume 50–150 W [40,41], indicating that the proposed AI-BOX achieves more than a tenfold improvement in power efficiency. Therefore, the proposed edge-based architecture demonstrates practical low-power performance suitable for intelligent parking management in embedded environments.

As shown in Figure 18b above, the memory usage of the proposed AI-BOX system averaged 0.93GB, representing an increase of 0.31 GB from the default state of 0.62 GB. Memory usage remained relatively stable, fluctuating between a minimum of 0.87 GB and a maximum of 0.99 GB, indicating effective management of memory resources. No memory leak issues were detected, and any temporary increases in memory usage quickly returned to normal levels. These temporary spikes primarily occurred during the processing of large data sets, demonstrating the system’s efficient handling capabilities.

As shown in Figure 18c above, the system’s average temperature was recorded at 39.5 °C, an increase of 6 °C from the baseline of 33.5 °C. While the temperature peaked at 45 °C, it remained well within the CPU’s safe operating range, alleviating concerns about overheating. Due to effective thermal management, the CPU temperature remained generally stable. Although there were brief spikes in temperature corresponding with increased CPU usage, the system promptly managed these increases to prevent any prolonged rise.

The evaluation results of the three metrics indicate that the proposed system operates efficiently, even with the limited resources of an edge device (AI-BOX). While there are temporary fluctuations in CPU and memory usage, overall CPU usage remains stable, and CPU temperature is maintained within an appropriate range. In summary, these results demonstrate that the system can operate reliably over extended periods in a real-world parking lot environment.

### 5.3. Evaluation of Power Consumption and System Resource Usage

According to the official Raspberry Pi 4 Model B Product Brief released by the Raspberry Pi Foundation (2023) [39], the device operates within a typical power envelope of 2.5 W to 9 W (5 V × 0.5–1.8 A) depending on system workload. Independent benchmarking reports, such as the Pi Dramble Wiki Benchmark [42], further specify that the board consumes approximately 2.7 W in an idle state and 6.4 W under full-core CPU load. Based on these observations, a utilization above roughly 80–90% CPU can be regarded as a high-load operating condition, typically corresponding to 6–7 W or more of total power consumption.

To estimate instantaneous power consumption as a function of CPU and memory utilization, the following linear approximation was applied:

(4) $$P_{\mathrm{est}}=2.8+0.037\;U_{\mathrm{cpu}}+0.004\;U_{\mathrm{mem}}\;$$

where $U_{\mathrm{cpu}}$ and $U_{\mathrm{mem}}$ represent the average utilization percentages of the CPU and memory, respectively. This model was derived from the measured power range of the target environment and provides a practical means to infer the device’s power behavior under various computational loads.

**Figure 19:**
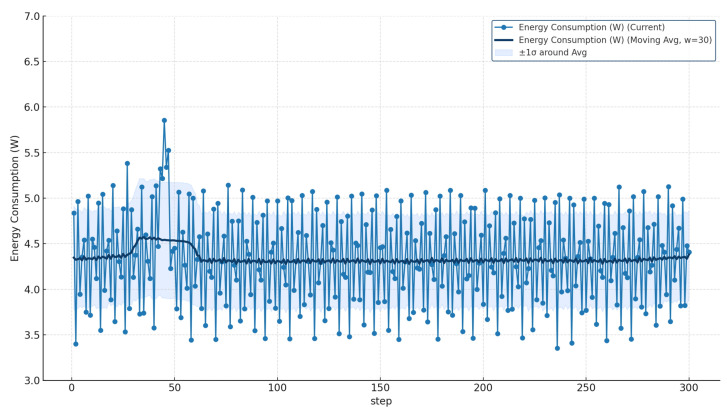
Estimated power consumption trend based on CPU and memory utilization.

When assuming that a load condition exists above 6 W, as illustrated in Figure 19 and Table 11, the proposed AI-BOX achieved a measured maximum power consumption of 5.86 W and a minimum of 3.36 W, confirming that it can operate stably within the power constraints of small-scale, low-power embedded boards.

These results confirm that the proposed AI-BOX configuration maintains stable and controllable power consumption within the embedded operational range. The estimated power usage remained well within the safe limits defined in the official specifications, demonstrating the system’s suitability for long-term, energy-efficient deployment in edge environments.

## 6. Conclusions

In this study, we proposed and implemented an outdoor parking space management system that utilizes edge computing technology and artificial intelligence. This system overcomes the limitations of centralized parking management, providing an efficient, real-time, and scalable alternative. The main contributions and achievements of this study are summarized as follows:

- We developed a distributed processing architecture centered on AI-BOX and designed an intuitive web-based monitoring and management interface to enhance system usability and efficiency.
- AI-BOX was developed on a low-power embedded board by deploying the YOLOv8 model, enabling real-time parking recognition at low cost.
- The system achieved 99% precision and 97% recall, demonstrating high recognition performance.
- Stable operation and reliability were validated through real-world parking lot tests, with efficient resource usage (CPU 31.5%, memory 0.93 GB).

The outdoor parking space management system based on the edge device (AI-BOX) proposed in this study facilitates efficient parking space management through high accuracy and real-time performance. By reducing network load and enabling real-time decisions through edge computing, the limitations of centralized systems are effectively resolved. Furthermore, the scalability and user convenience of the system have been significantly enhanced through a modular design and a web-based interface.

In addition to recognition accuracy and resource stability, latency evaluation confirmed the feasibility of embedded deployment under real-time constraints. At the median level (p50), YOLOv8 achieved approximately 195 ms, which is about 73 ms faster than YOLOv12 (268 ms), corresponding to a 27% improvement. At the p95 level, both models converged above 400 ms, with YOLOv12 recording approximately 415 ms and YOLOv8 approximately 405 ms, resulting in only a 10 ms (≈2.5%) difference. These results indicate that in low-power embedded environments for parking management applications, the latest model (YOLOv12) does not necessarily provide a competitive advantage; instead, a lighter model such as YOLOv8, which offers balanced efficiency and accuracy, can be more suitable. Taken together, the proposed system demonstrates robust accuracy, efficient resource utilization, and practical latency performance in real-world parking environments.

Future research plans aim to enhance and develop the system in the following directions:

- Improving recognition performance in various weather conditions and lighting environments to enhance the system’s stability;
- Enhancing system scalability by improving the capability to efficiently integrate and manage multiple cameras in large parking lots;
- Expanding the comparative evaluation of object detection models. In this study, YOLOv8 and YOLOv12 were benchmarked under identical embedded environments, and future work will extend this comparison to upcoming versions (YOLOv9, YOLOv10, and YOLOv11) to analyze accuracy speed trade-offs and embedded optimization potential’
- Conducting comparative experiments with other state-of-the-art object detection models, such as Faster R-CNN, MobileNet-SSD, and EfficientDet, and performing validation on public datasets, including COCO and Pattern Analysis, Statistical Modeling and Computational Learning Visual Object Classes (Pascal VOC), to evaluate the model’s generalization capability.

The results of this study are expected to significantly enhance the efficiency and convenience of outdoor parking lot management. Specifically, compared to conventional ultrasonic or in-ground sensor-based parking management systems, the proposed system offers higher adaptability to changes in parking spaces and provides significant advantages in terms of initial deployment cost. Additionally, this technology has the potential for expansion into various fields, including traffic flow analysis and vehicle security, playing a crucial role in the development of intelligent transportation systems.

## Figures and Tables

**Table 1:** Comparison of parking occupancy monitoring methods.

Method	CAPEX	Characteristics
**In-ground Sensors [17,18]**	$250–$800/slot	Robust detection using embedded ground sensors, but cost scales linearly with each slot
**On-surface IoT Sensors [19]**	$300–$500/slot	Easy wireless installation, but battery lifecycle increases maintenance needs
**Centralized Camera System [20]**	$100–$400/camera	Slot-level monitoring via server inference, but scalability is limited by network load

**Table 2:** Performance comparison of *AIOHTTP* and other Python web frameworks (Source: Adapted from [36]).

Framework	Request Throughput (req/s)	Average Response Time (ms)
AIOHTTP	10,000	5
Flask	5000	10
Django	4000	12

**Table 3:** Main Functions of the Edge Device (AI-BOX) Module in the Proposed System.

Module	Description
Video Input Module	Efficiently receives and decodes RTSP [37,38] streams using FFmpeg.
Video Processing Module	Preprocesses the input video and converts it into a form suitable for object detection.
Object Detection Module	Utilizes the YOLOv8 algorithm to quickly and accurately detect vehicle objects.
Parking Space Analysis Module	Compares detected vehicle objects with predefined parking space information to determine occupancy.
Data Management Module	Stores and manages the analysis results.
Web Interface Module	Provides processed data via API and handles communication with the administrator dashboard.

**Table 4:** AI-BOX Hardware Specifications.

Component	Specification
CPU	Broadcom BCM2711, Quad core Cortex-A72 (ARM v8) 64-bit SoC @ 1.5 GHz
GPU	Broadcom VideoCore VI, 500 MHz
RAM	4 GB LPDDR4-2400 SDRAM
Network	2.4 GHz and 5.0 GHz IEEE 802.11ac Wireless, Gigabit Ethernet
Storage	64 GB Micro-SD Card, 500 GB External SSD (Solid-State Drive) (USB (Universal Serial Bus) 3.0 connection)
Power	5 V DC via USB-C connector (5 V/3 A)
Interface	2 × USB 3.0 ports, 2 × USB 2.0 ports, 1 × CSI (Camera Serial Interface) camera port, 1 × micro-HDMI port

**Table 5:** Final Vehicle Object Classification Training Dataset.

Dataset Classification	Occupied Parking Spaces	Empty Parking Spaces
Training Data	5079	3271
Validation Data	555	327
Test Data	2248	2211
**Total**	**7882**	**5809**

**Table 6:** YOLOv8n Model Training Hyperparameters.

Parameter	Value
Input image size	640 × 640
Batch size	16
Number of epochs	20
Learning rate	0.01 (cosine annealing schedule)
Optimizer	Stochastic Gradient Descent (SGD)
Momentum	0.937
Weight decay	0.0005
Warmup epochs	3
Confidence threshold	0.5
IoU threshold (NMS)	0.7
Augmentation techniques	Random rotation, flipping, brightness adjustment

**Table 7:** Key Screens and Features of the Monitoring Web Service.

Screens	Main Features
Login Screen and Password Change	User authentication Password change feature
Camera Information Settings	Enter RTSP URL and Web URL Check camera connection status Display live video feed Control camera PTZ (Pan-Tilt-Zoom)
Parking Space Settings	Add/modify/delete parking space areas Set parking space information (name, count, direction, number, type) Apply and save settings
VMS (Video Management System) Event Transmission	Event activation settings Enter VMS URL Set transmission interval Set special events
Event Log Delivery	Set event log delivery Set delivery interval and recipients

**Table 8:** Comparison of performance between YOLOv8 and YOLOv12.

Model	Class	Precision	Recall	mAP@0.5	mAP@0.5:0.95
YOLOv8	All	0.991	0.975	0.992	0.872
	Car	0.986	0.964	0.987	0.826
	Disabled	0.993	0.978	0.994	0.880
	Empty	0.993	0.983	0.994	0.911
YOLOv12	All	0.991	0.975	0.992	0.872
	Car	0.988	0.972	0.988	0.826
	Disabled	0.996	0.978	0.994	0.878
	Empty	0.994	0.986	0.995	0.913

**Table 9:** Benchmark comparison of YOLOv8 and YOLOv12 (first 50 runs excluded).

Metric	YOLOv8	YOLOv12
Iterations	250	250
Avg Preprocess Time (ms)	1.78 ± 1.78	1.93 ± 2.04
Avg Inference Time (ms)	224.53 ± 92.82	254.35 ± 103.80
Avg Postprocess Time (ms)	0.49 ± 0.94	0.38 ± 0.67
Avg Total Time (ms)	226.81 ± 93.37	256.67 ± 104.52
Min Total Time (ms)	118.48	135.57
Max Total Time (ms)	504.05	485.39

**Table 10:** Evaluation Results of System Resource Usage.

Metric	Maximum	Minimum	Average	Baseline
CPU Usage	72.5%	4.8%	31.5%	3.2%
Memory Usage	0.99 GB	0.87 GB	0.93 GB	0.62 GB
CPU Temperature	45 °C	36.6 °C	39.5 °C	33.5 °C

**Table 11:** Evaluation Results of Energy Consumption.

Metric	Maximum	Minimum	Average	Baseline
Energy Consumption	5.86 W	3.36 W	4.42 W	3.35 W

## Data Availability

The trained AI model and source code of the proposed AI-BOX system are not publicly available due to commercial restrictions and intellectual property protection. However, summarized experimental configurations, parameter settings, and representative results can be shared upon reasonable request for academic research purposes.

## References

[1] Biyik C., Allam Z., Pieri G., Moroni D., O’Fraifer M., O’Connell E., Olariu S., Khalid M. (2021). Smart Parking Systems: Reviewing the Literature, Architecture and Ways Forward. Smart Cities.

[2] Lin T., Rivano H., Le Mouël F. (2017). A survey of smart parking solutions. IEEE Trans. Intell. Transp. Syst..

[3] Litman T. (2024). Parking Management: Strategies, Evaluation and Planning. https://www.vtpi.org/park_man.pdf.

[4] Victoria Transport Policy Institute (2025). Comprehensive Parking Supply, Cost and Pricing Analysis. https://www.vtpi.org/pscp.pdf.

[5] Paidi V., Fleyeh H., Håkansson J., Nyberg R.G. (2018). Smart parking sensors, technologies and applications for open parking lots: A review. IET Intell. Transp. Syst..

[6] Channamallu S.S., Kermanshachi S., Rosenberger J.M., Pamidimukkala A. (2023). A review of smart parking systems. Transp. Res. Procedia.

[7] Barriga J.J., Sulca J., León J.L., Ulloa A., Portero D., Andrade R., Yoo S.G. (2019). Smart parking: A literature review from the technological perspective. Appl. Sci..

[8] Ala’anzy M.A., Abilakim A., Zhanuzak R., Li L. (2025). Real time smart parking system based on IoT and fog computing evaluated through a practical case study. Sci. Rep..

[9] Mackowski D., Bai Y., Ouyang Y. (2015). Parking space management via dynamic performance-based pricing. Transp. Res. Procedia.

[10] Sarker V.K., Gia T.N., Ben Dhaou I., Westerlund T. (2020). Smart parking system with dynamic pricing, edge-cloud computing and lora. Sensors.

[11] Hassoune K., Dachry W., Moutaouakkil F., Medromi H. Smart parking systems: A survey. Proceedings of the 2016 11th International Conference on Intelligent Systems: Theories and Applications (SITA).

[12] Mainetti L., Patrono L., Sergi I. A survey on indoor positioning systems. Proceedings of the 2014 22nd International Conference on Software, Telecommunications and Computer Networks (SoftCOM).

[13] Mao Y., You C., Zhang J., Huang K., Letaief K.B. (2017). A survey on mobile edge computing: The communication perspective. IEEE Commun. Surv. Tutor..

[14] Anagnostopoulos T., Zaslavsky A., Kolomvatsos K., Medvedev A., Amirian P., Morley J., Hadjieftymiades S. (2017). Challenges and opportunities of waste management in IoT-enabled smart cities: A survey. IEEE Trans. Sustain. Comput..

[15] Ultralytics (2023). YOLOv8 Documentation. https://docs.ultralytics.com/.

[16] Khanna A., Anand R. IoT based smart parking system. Proceedings of the 2016 International Conference on Internet of Things and Applications (IOTA).

[17] Moini N., Hill D., Gruteser M. (2012). Impact Analyses of Curb-Street Parking Guidance System on Mobility and Environment.

[18] Zakharenko R. (2019). The economics of parking occupancy sensors. Econ. Transp..

[19] U.S. Department of Transportation, ITS JPO (2020). ITS Knowledge Resources: Cost Record 2020-SC00464..

[20] Fahim A., Hasan M., Chowdhury M.A. (2021). Smart parking systems: Comprehensive review based on various aspects. Heliyon.

[21] Krizhevsky A., Sutskever I., Hinton G.E. (2012). Imagenet classification with deep convolutional neural networks. Adv. Neural Inf. Process. Syst..

[22] Redmon J., Divvala S., Girshick R., Farhadi A. You only look once: Unified, real-time object detection. Proceedings of the IEEE Conference on Computer Vision and Pattern Recognition.

[23] Howard A.G., Zhu M., Chen B., Kalenichenko D., Wang W., Weyand T., Andreetto M., Adam H. (2017). Mobilenets: Efficient convolutional neural networks for mobile vision applications. arXiv.

[24] Iandola F.N., Han S., Moskewicz M.W., Ashraf K., Dally W.J., Keutzer K. (2016). SqueezeNet: AlexNet-level accuracy with 50x fewer parameters and <0.5 MB model size. arXiv.

[25] Zhang X., Zhou X., Lin M., Sun J. Shufflenet: An extremely efficient convolutional neural network for mobile devices. Proceedings of the IEEE Conference on Computer Vision and Pattern Recognition.

[26] Jocher G., Chaurasia A., Qiu J. (2023). YOLO by Ultralytics (Version 8.0.0) [Computer Software]. https://github.com/ultralytics/ultralytics.

[27] Lin T.Y., Maire M., Belongie S., Hays J., Perona P., Ramanan D., Dollár P., Zitnick C.L. (2014). Microsoft coco: Common objects in context. Proceedings of the European Conference on Computer Vision.

[28] Nwankpa C. (2018). Activation functions: Comparison of trends in practice and research for deep learning. arXiv.

[29] Xiong Y., Liu H., Gupta S., Akin B., Bender G., Wang Y., Kindermans P.J., Tan M., Singh V., Chen B. Mobiledets: Searching for object detection architectures for mobile accelerators. Proceedings of the IEEE/CVF Conference on Computer Vision and Pattern Recognition.

[30] Liu S., Zha J., Sun J., Li Z., Wang G. (2023). EdgeYOLO: An Edge-Real-Time Object Detector. arXiv.

[31] Wang A., Chen H., Liu L., Chen K., Lin Z., Han J. (2024). Yolov10: Real-time end-to-end object detection. Adv. Neural Inf. Process. Syst..

[32] Zhang H., Hu W., Wang X. (2022). EdgeFormer: Improving Light-weight ConvNets by Learning from Vision Transformers. arXiv.

[33] (2025). Asynchronous HTTP Client/Server for Asyncio and Python. https://docs.aiohttp.org/.

[34] Rachapudi A., Parimi P., Alluri S. (2020). Performance Comparison of Applications with and without Web Frameworks. Int. J. Adv. Trends Comput. Sci. Eng..

[35] Paterson C. (2020). Async Python is Not Faster. https://calpaterson.com/async-python-is-not-faster.html.

[36] Bednarz B., Miłosz M. (2025). Benchmarking the performance of Python web frameworks. J. Comput. Sci. Inst..

[37] Rao A., Lanphier R., Schulzrinne H. *Real Time Streaming Protocol (RTSP)*; RFC 2326; RFC Editor: 1998. https://www.rfc-editor.org/info/rfc2326.

[38] Schulzrinne H., Rao A., Lanphier R., Westerlund M., Stiemerling M. *Real-Time Streaming Protocol Version 2.0*; RFC 7826; RFC Editor: 2016. https://www.rfc-editor.org/info/rfc7826.

[39] Raspberry Pi Foundation (2023). Raspberry Pi 4 Model B Product Brief—Power and Performance Specifications. https://www.raspberrypi.com/documentation/computers/raspberry-pi.html.

[40] NVIDIA Corporation (2023). Jetson AGX Xavier Series Data Sheet. https://developer.nvidia.com/embedded/jetson-agx-xavier.

[41] Google Cloud (2023). Energy Consumption of Cloud GPU Instances. https://cloud.google.com/compute/docs/gpus.

[42] Wiki P.D. (2024). Power Consumption Benchmarks for Raspberry Pi 4 Model B. https://www.pidramble.com/wiki/benchmarks/power-consumption.

